# Public mental health – a challenge for local communities and research

**DOI:** 10.1017/S0033291724001478

**Published:** 2024-10

**Authors:** Stefan Priebe, Ulrich Reininghaus

**Affiliations:** 1 Centre for Mental Health Research, City, University of London, London, UK; 2 Central Institute of Mental Health, University of Heidelberg, Mannheim, Germany

**Keywords:** local communities, mental health, policy, public health, research, social determinants, voluntary, weak ties

## Abstract

Common approaches for improving the mental health of the population in general and of vulnerable groups in particular include policies to address social determinants and the expansion of professional health services. Both approaches have substantial limitations in practice. A more promising option is actions that utilize resources that either already exist or can easily be generated in local communities. Examples are provided for various local initiatives with the potential to facilitate helpful interactions and relationships that are likely to benefit the mental health of significant parts of the population. Developing and implementing such initiatives is a challenge to communities, while their evaluation may require innovative methods in research.

Improving the mental health of the population is increasingly seen as a major societal challenge. Improvements should benefit the whole population and particularly support vulnerable groups, who are at higher risk of suffering from poor mental health (Dykxhoorn et al., 2022; Frohlich & Potvin, 2008; Reininghaus et al., 2022). While there are wide-spread calls for action to help those groups, it remains unclear what exactly should be done ideally and what can be done realistically.

Two approaches are commonly considered: targeting social determinants of poor mental health and strengthening professional health services for providing prevention and treatment. Both approaches look promising in theory but are limited in practice.

The most important social determinants of mental disorders are well-researched and widely known (Dykxhoorn et al., 2022; Marmot, Allen, Bell, Bloomer, & Goldblatt, 2012). Upbringing adversity, socio-economic hardship, poor education, experience of war and torture, homelessness, unemployment, and income inequality have all been documented to contribute to a higher prevalence of mental disorders. Considering the overwhelming evidence, it appears important for professional groups to raise a voice in the public debate and point to the need to target and influence those factors. Sharing the evidence about social determinants is an ethical obligation for all experts, and calling for political action for addressing the determinants may give professional groups credibility and societal relevance (Priebe, 2015). However, any substantial change in major social determinants requires effective political actions and such actions are beyond what professional groups can directly achieve.

Professional mental health services cannot be expected to have a substantial impact on mental health on a population level either. In most countries, these services are overstretched already and there are limitations to plans for expanding them: (A) More services require more funding that will be hard, if not impossible, to generate. (B) Even if there was more funding, in many countries there are not enough qualified health professionals to take up additional posts. (C) Even if there were enough funding and enough staff, conventional treatments – medication or psychological therapies – are not appealing to everyone, and particularly many young people do not engage with such treatments. (D) Even if young people engage in conventional psychological therapies, these therapies have an only limited effect on a group level, and even the limited effect is often not lasting (Cuijpers et al., 2021). And (E), even if those treatments would be more effective, they inevitably come with the inherent message that the affected individual cannot cope with their crisis on their own and that they need professional support to overcome mental distress, a message that particularly in young people may undermine confidence, and have a negative effect on how they can deal with crises during the rest of their lives. One can add to these limitations significant general concerns about the overmedicalization of mental distress in our societies (Economist Intelligence Unit N.A. Incorporated, 2023).

What can be done, when fundamental political action for improving major social determinants may not be forthcoming and expanding health services is neither a realistic nor a very useful solution? One option is to utilize resources that already exist or can easily be generated in local communities and that have a potential benefit for the mental health of everyone living in the given community, including marginalized and vulnerable groups.

Many communities already have a range of rich resources for improving mental health. There are plenty of voluntary organizations that provide activities in the community that can engage people suffering from mental distress and others at risk of developing it. These activities include sports and arts, which can appeal to many who would not necessarily engage with conventional psychological treatments (Hidalgo-Padilla et al., 2022). Sport organizations exist all over the world, and evidence suggests that particularly team sports may reduce anxiety and depression among young people. While these organizations will commonly aim to grow the next sports star, they are aware that most participants in sport will not acquire outstanding skills. Thus, the organizations also aim to develop team spirit, raise confidence, and facilitate personal growth in all those who participate on different levels of skills (Hoffmann, Barnes, Tremblay, & Guerrero, 2022).

A similar resource is community arts organizations. They use different modalities including visual arts, music, dance, and theatre to engage people in local communities. Like sports clubs, these organizations may aspire to grow outstanding talent but also promote personal experiences, interactions with others, and dealing with emotions (Goodman-Casanova, Guzman-Parra, Mayoral-Cleries, & Cuesta-Lozano, 2023). Sport and arts organizations vary widely depending on their specific activities, target groups, leadership, funding, size, set up, and context. However, in many of those organizations there is an interest in helping their participants to avoid or overcome mental distress. In a rather informal and variable way, they may already provide such help in an effective manner. At the same time, staff can feel overwhelmed when participants show signs of mental distress. They may lack the time or confidence to deal with such problems and would appreciate support and guidance to help those participants better who show signs of mental distress. The support may require regular meetings for peer support within staff teams, networking, training in principles of how to conduct conversations with distressed people, and possibly even limited external supervision. While some of this can require additional funding, the amounts will be very limited and might be generated through public sources or social prescribing (Bickerdike, Booth, Wilson, Farley, & Wright, 2017), acknowledging the function of the organizations for the mental health of the community. Further resources may be mobilized through other voluntary organizations that provide support for marginalized groups and opportunities for very different activities. For instance, evidence suggests that religious communities can provide strong cohesion and support with a positive influence on the mental health of their members (Debnam, Holt, Clark, Roth, & Southward, 2012).

The resources mentioned so far already exist and just need utilizing and promoting to strengthen their benefit for public mental health. Other resources in form of supportive interactions in the community can be established but need new initiatives to be set up. Here are a few examples:

In most parts of the world the population is aging, and an increasing number of retired people suffer from social isolation. Some of them cannot continue in their original profession but may be available for local activities on a flexible part-time basis, possibly only for a few hours per week. That resource may be used to provide local childcare for young families which otherwise would not exist or be very expensive. Such an arrangement can provide meaningful and feasible occupation for the elderly, help to overcome their isolation with wide-ranging physical and mental health benefits (Choi, Irwin, & Cho, 2015), foster intergenerational contacts and community cohesion, and facilitate raising children in the light of low birth rates in most parts of the world.

In rural areas, communities can run community shops and community pubs which bring people together, make them share a task, create collaboration, provide a sense of common success, and promote interactions that may help to prevent and overcome mental distress.

In urban areas, simple parklets can increase the chance that people pause and talk with each other. More ambitious projects are the establishment of blocks of a few streets with limited through traffic, enhanced green space, reduced air pollution, and the provision of most required amenities, such as the ‘superilles’ in Barcelona or the ‘Kiezblocks’ in Berlin (Palència et al., 2020). They help people to spend time in public spaces and engage in interactions and relationships. Some of these interactions are likely to provide direct emotional and practical support. Others may help to establish networks of various flexible and loose relationships, in the sense of the ‘weak ties’ that in sociology have been suggested as a central resource for people already 50 years ago (Granovetter, 1973). Weak ties widen the options for exchange, for further contacts, and for both providing and finding variable support when needed (Collins, Hagerty, Quoidbach, Norton, & Brooks, 2022; Moreton, Kelly, & Sandstrom, 2023).

These few examples all describe win–win situations, requiring little or no financial investment. Not only can they improve the mental health of the population but they may also have wider societal benefits such as better physical health and more favorable quality of life. What is required to turn these ideas into reality?

Depending on the national context and regional systems of political authority, some tweaks to legislation and national or regional policies may be helpful or even necessary. However, the umbrella perspective that combines all of the above-mentioned initiatives is the local community. While the activities may not require much funding, they do need the initiative, energy, and skills of individuals and small groups that can drive and sustain them. This can be provided only on a local level, and how best to do it will depend on factors that vary substantially between and within countries. The enthusiasm of people to engage in local communities and the type of activities they favor cannot be prescribed and will not be consistent across different contexts.

The effect of such local initiatives on the mental health of the population is rather indirect and likely to vary for different groups. To argue for the promotion of such initiatives in the interest of public mental health and for designing them effectively, research evidence on different models, costs, experiences, and benefits would be helpful. However, current research models and processes may struggle to meet that challenge. One reason for this is the protracted nature of academic research. Such research requires funding, and the preparation, submission, and processing of applications for funding can take up to a year and more. The bureaucracy of academic research leads to further delays and prolongations, and the costs are often high. Once the studies have started, they can take several years until completion, by which time the ideas for local initiatives and the political landscape for implementing them may have already changed. However, the rules and practice of academic research are not the only barriers. There is also a need to go beyond the currently established research designs that are commonly funded by grant awarding bodies. Cluster randomized controlled trials may be appropriate in some cases. In these trials, full areas with the affected individuals living in the given area are randomized to an experimental or control condition (Eldridge & Kerry, 2012). This can be done with step wedge designs, in which the intervention is rolled out successively so that areas beginning the intervention later can learn from the ones that started earlier. Such experimental studies can be used to test whether some of the interventions make a real difference to the populations in different areas. However, community initiatives tend to be variable, fast evolving, and fluid; and they are frequently shaped by charismatic individuals and depend on specific characteristics of the context. This makes it difficult to operationalize and replicate them in the same form in different areas. This is a limitation to experimental research designs as such designs require some clear and replicable specification of what exactly is being tested. Thus, more flexible observational research studies are required in addition to transparent routine documentations of what is happening. Such research needs to identify helpful and less helpful components and provide data about the forms, uptake, experiences, and outcomes of existing and new initiatives so that others can learn from them to shape and improve their own plans and actions. The collection and analysis of already available data, e.g. in social media, may open up new opportunities for such research.

Improving the mental health of the population may be achieved through actions that help all people, including vulnerable groups, to utilize and benefit from social resources. This is a challenge for local communities. It also is a challenge to the international research community that should provide evidence supporting and guiding new policies and initiatives.
